# Double-Sine-Wave Quadri-Pulse Theta Burst Stimulation of Precentral Motor Hand Representation Induces Bidirectional Changes in Corticomotor Excitability

**DOI:** 10.3389/fneur.2021.673560

**Published:** 2021-06-28

**Authors:** Nikolai H. Jung, Bernhard Gleich, Norbert Gattinger, Anke Kalb, Julia Fritsch, Elisabeth Asenbauer, Hartwig R. Siebner, Volker Mall

**Affiliations:** ^1^School of Medicine, Social Pediatrics, Technical University of Munich, Munich, Germany; ^2^Munich School of Bioengineering (MSB), Technical University of Munich, Garching, Germany; ^3^Danish Research Center for Magnetic Resonance, Centre for Functional and Diagnostic Imaging and Research, Copenhagen University Hospital, Amager and Hvidovre, Copenhagen, Denmark; ^4^Institute for Clinical Medicine, Faculty of Medical and Health Sciences, University of Copenhagen, Copenhagen, Denmark; ^5^Department of Neurology, Copenhagen University Hospital Bispebjerg and Frederiksberg, Copenhagen, Denmark

**Keywords:** transcranial magnetic stimulation, double-sine pulses, non-invasive brain stimulation, neuronal plasticity, corticospinal excitability, human primary motor cortex, long-term potentiation, long-term depression

## Abstract

Neuronal plasticity is considered to be the neurophysiological correlate of learning and memory and changes in corticospinal excitability play a key role in the normal development of the central nervous system as well as in developmental disorders. In a previous study, it was shown that quadri-pulse theta burst stimulation (qTBS) can induce bidirectional changes in corticospinal excitability (1). There, a quadruple burst consisted of four single-sine-wave (SSW) pulses with a duration of 160 μs and inter-pulse intervals of 1.5 ms to match I-wave periodicity (666 Hz). In the present study, the pulse shape was modified applying double-sine-waves (DSW) rather than SSW pulses, while keeping the pulse duration at 160 μs. In two separate sessions, we reversed the current direction of the DSW pulse, so that its second component elicited either a mainly posterior-to-anterior (DSW PA-qTBS) or anterior-to-posterior (DSW AP-qTBS) directed current in the precentral gyrus. The after-effects of DSW qTBS on corticospinal excitability were examined in healthy individuals (*n* = 10) with single SSW TMS pulses. For single-pulse SSW TMS, the second component produced the same preferential current direction as DSW qTBS but had a suprathreshold intensity, thus eliciting motor evoked potentials (PA-MEP or AP-MEP). Single-pulse SSW TMS revealed bidirectional changes in corticospinal excitability after DSW qTBS, which depended on the preferentially induced current direction. DSW PA-qTBS at 666 Hz caused a stable increase in PA-MEP, whereas AP-qTBS at 666 Hz induced a transient decrease in AP-MEP. The sign of excitability following DSW qTBS at I-wave periodicity was opposite to the bidirectional changes after SSW qTBS. The results show that the pulse configuration and induced current direction determine the plasticity-effects of ultra-high frequency SSW and DSW qTBS at I-wave periodicity. These findings may offer new opportunities for short non-invasive brain stimulation protocols that are especially suited for stimulation in children and patients with neurological or neurodevelopmental disorders.

## Introduction

Synaptic plasticity is considered to be the neurophysiological correlate of learning and memory and changes in corticospinal excitability play a key role in the normal development of the central nervous system as well as in developmental disorders (2, 3). Regular or patterned repetitive transcranial magnetic stimulation (rTMS) of the precentral motor representations can induce lasting bidirectional changes in corticomotor excitability revealed by a lasting change in the mean amplitude of the motor evoked potential (MEP). This change in corticomotor excitability is attributed to changes in synaptic efficacy in the stimulated corticospinal system and therefore referred to as long-term potentiation (LTP)-like or long-term depression (LTD)-like plasticity (4). LTP and LTD are supposed to be the neurophysiological correlate of learning and memory (5, 6).

Extending the classic rTMS protocols, we recently introduced a novel quadri-pulse theta-burst stimulation (qTBS) protocol (1). The burst protocol consisted of four single-sine-wave (SSW) pulses which were given at an ultra-high pulse repetition rate of 666 Hz and a burst repetition rate of 5 Hz. We chose a within-burst repetition rate of 666 Hz to mimic the periodicity of descending I-waves that are generated by TMS (7). Depending on the preferential current direction, our novel SSW qTBS protocol consistently induced lasting bidirectional changes in corticospinal excitability in the human precentral motor hand representation (1).

The SSW qTBS protocol recombined two established patterned rTMS protocols, namely theta-burst stimulation (TBS) and quadri-pulse stimulation (QPS) that were previously demonstrated to effectively induce changes in corticospinal excitability by primarily targeting two different mechanisms (8, 9). While TBS is Ca^2+^-dependent with a frequency leading to an optimal post-synaptic Ca^2+^ influx that is required for LTP- and LTD–like plasticity (10, 11), QPS effectively induces synaptic plasticity at interstimulus intervals, mimicking the rhythmic pattern of multiple descending volleys (so-called I-wave rhythmicity) that can be recorded in the corticospinal tract (7, 9, 12). These descending volleys are composed of multiple excitatory and inhibitory (GABAergic) neurons and axons of different sizes, location, orientation, and function and activate presynaptic neural elements to the corticospinal cell (7, 13).

In the present study, we modified our novel qTBS with ultra-high within-burst frequency bursts. We altered the pulse configuration using double-sine-wave (DSW) pulses rather than single-sine-wave (SSW) while keeping the pulse duration constant. DSW pulses were generated by a new stimulation device designed and built by B.G. and N.G., Munich School of Bioengineering, Technical University of Munich, Garching, Germany, which enabled us to apply DSW pulses at ultra-high pulse repetition rates that mimic I-wave periodicity (i.e., 666 Hz). We hypothesized that DSW pulses would be more effective in inducing changes in corticomotor excitability. The use of DSW pulses was motivated by our own findings that concatenated full-sine cycles decrease the threshold of local excitability with a maximum at two sine (14). We assumed that these polyphasic TMS pulses at a sequence within the medium frequency band of 1–300 kHz may improve the effectiveness of high frequency patterned rTMS protocols such as qTBS by summation of subthreshold excitations within one pulse cycle, which is analogous to the so-called “Gildemeister effect” at peripheral nerves (15). This effect describes that highfrequency pulse cycles do not necessarily generate action potentials themselves, but subthreshold excitations of subsequent pulse phases may be integrated (15, 16). This has been shown in animal studies where electrical peripheral nerve stimulation of reversed DSW pulses resulted in a summation of the excitatory effect (16). Moreover, the stimulation effect of reverse DSW pulses depended on the sequence of polarity and the value of the membrane potential. At hyperpolarization of the membrane, the initial negative DSW pulse was more effective, whereas at depolarization of the membrane, the initial positive DSW was more effective (16). The coupling of full-sine pulses resulted in changes in the threshold voltage for nerve excitation (16).

In the present study, we aimed to investigate how DSW qTBS generating ultra-high frequency bursts at I-wave periodicity (666 Hz) shapes corticomotor excitability. For DSW pulses, we selected an initial current direction of the DSW to ensure that the second component of the DSW would elicit either a preferentially posterior-to-anterior (DSW PA-qTBS) or anterior-to-posterior (DSW AP-qTBS) directed current in the precentral gyrus. The aftereffects of DSW qTBS on corticomotor excitability were examined using single SSW TMS pulses as in our previous study in order to facilitate comparability of results (1). Single-pulse SSW TMS produced the same preferential current direction in the precentral gyrus as DSW qTBS but had a suprathreshold intensity, thus eliciting motor evoked potentials (PA-MEP or AP-MEP). We hypothesized that DSW pulses of the same total pulse length of 160 μs as used for SSW qTBS may introduce stronger plasticity-inducing effects.

## Materials and Methods

### Participants

In DSW qTBS experiments, ten healthy volunteers (5 women, 5 men) aged 20–37 years (median age 22.5 years; SD 4.85) participated in the study after giving written informed consent. In SSW qTBS experiments, twelve healthy volunteers (7 women, 5 men) aged 18–36 years (median age 23.5 years; SD 4.45) participated. Seven volunteers (4 women, 3 men, aged 20–37 years; median age 22 years; SD 5.55) took part in SSW qTBS and DSW qTBS experiments. The study was approved by the local Ethics Committee of the Technical University of Munich, Faculty of Medicine (vote 5423/12) and carried out according to the Code of Ethics of the World Medical Association (Declaration of Helsinki). Eight participants were right-handed and two were left-handed according to the Edinburgh Handedness Inventory (17). A structured interview according to existing guidelines revealed none of the participants as having either a history of neurological or psychiatric illnesses, nor meeting any exclusion criteria concerning the safety of TMS (18, 19). When comparing single-sine and double-sine data, we analyzed the same participants that took part in our previously published study (1).

### Experimental Procedures

**Figure 1:**
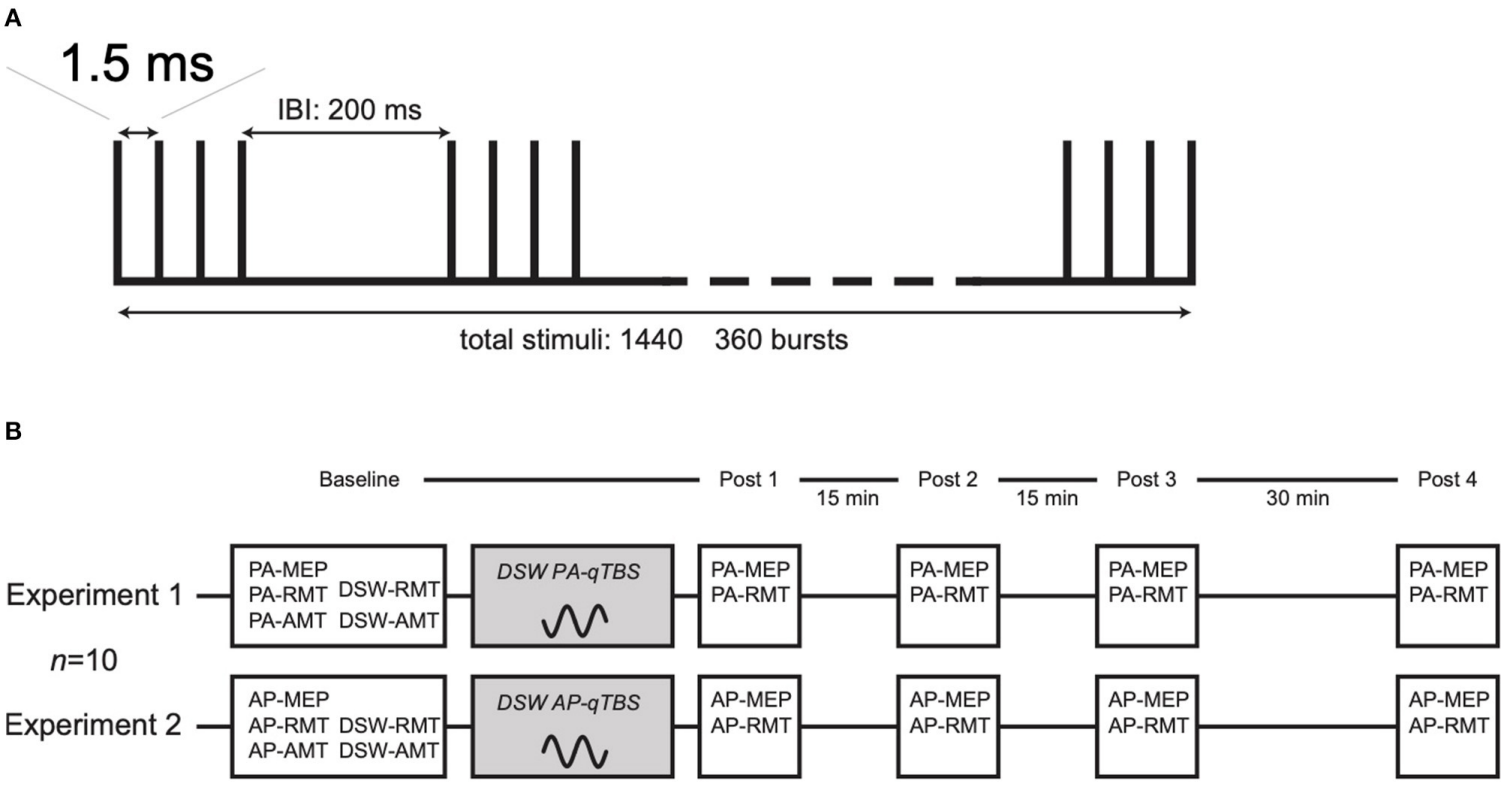
Schematic drawing of DSW and SSW qTBS pulse sequence and experimental procedures **(A)** qTBS consists of 360 trains of DSW or SSW TMS pulses. Each train consists of magnetic pulses delivered at interstimulus intervals of 1.5 ms resulting in a total of 1,440 stimuli. Trains were repeated every 200 ms. **(B)** Experimental procedures, a timeline of experiments and number of participants for DSW Experiments 1 and 2. Experimental procedures and timelines resembled those introduced in our previous study for SSW qTBS (1). In Experiment 1, the interstimulus interval (ISI) of each double-sine-wave (DSW) qTBS pulse was set to 1.5 ms (666 Hz) to test the potential I-wave frequency-dependent patterns of DSW qTBS with an effective induced current in the precentral gyrus flowing from posterior to anterior (PA). In Experiment 2, the direction of the induced current was changed to an anterior-posterior direction (AP). For single-sine-wave (SSW) pulse TMS, cycles of corresponding current direction were applied. Details of waveforms and preferentially induced current directions are presented in Figure 2. The prefix PA and AP indicates the respective current direction in the precentral gyrus. MEP, motor evoked potential; RMT, resting motor threshold; AMT, active motor threshold; qTBS, quadri-pulse theta burst stimulation; DSW-RMT, resting motor threshold with double-sine-wave pulses; DSW-AMT, active motor threshold with double-sine-wave pulses; PA, posterior-anterior; AP, anterior-posterior.

**Figure 2:**
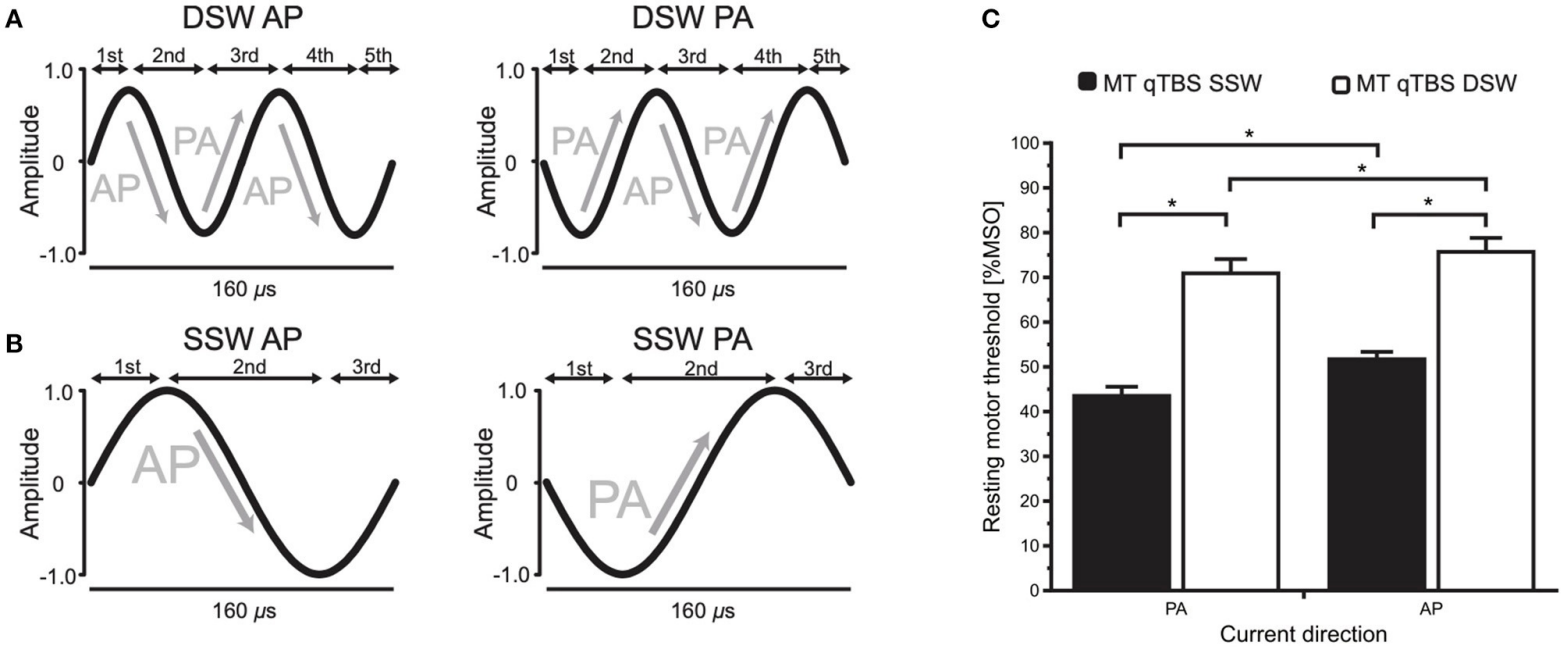
qTBS pulse sequence and current waveforms for double-sine-wave (DSW) and single-sine-wave (SSW) stimuli. **(A)** DSW pulses in PA and AP directions had a biphasic current waveform with 5 components with 160 μs total pulse duration. Current directions always refer to the electrical current produced by the second component of the DSW or SSW pulse in M1-HAND. **(B)** SSW pulses in PA and AP directions with 3 components and 160 μs total pulse duration were used for assessing corticomotor excitability. **(C)** Comparison of rMT data revealed by single pulse TMS using SSW and DSW pulses. RMT was significantly higher with DSW as compared to SSW pulses in PA- and AP-current directions (*post hoc t*-test: *p* = 0.02). Asterisks (^*^) indicate significant differences between measurements (*p* < 0.05). Error bars display the standard error of the mean (S.E.M.). qTBS, quadri-pulse theta burst stimulation; SSW, single-sine-wave; DSW, double-sine-wave; ISI, interstimulus interval; MEP, motor evoked potential; MSO, maximum stimulator output; IBI, interburst interval; AP, anterior-posterior; PA, posterior-anterior.

The experimental procedures closely resembled the procedures we had used previously for SSW qTBS (1). To investigate the direction dependency, the experiments were performed in PA (Experiment 1) and AP (Experiment 2) direction. A schematic drawing of the qTBS paradigm and a detailed timeline of Experiments 1 and 2 are depicted in Figures 1A,B. For DSW qTBS, the ISI of each pulse was set to 1.5 ms (666 Hz) to match I-wave periodicity (1, 12). Each DSW qTBS pulse consisted of two full-sine cycles with 80 μs duration, respectively, resulting in a total stimulus duration of 160 μs with a sine-frequency of 12.5 kHz (Figure 2A). We defined the direction of a DSW pulse according to which current direction is produced by its second component in the precentral gyrus. A DSW pulse has a PA direction if the second component of the pulse produces a posterior-to-anterior current in the precentral gyrus. Conversely, a DSW pulse has an AP direction, if its second phase produces an anterior-to-posterior current in the precentral gyrus (Figure 2A). The same nomenclature was used for SSW qTBS (Figure 2B) (1). To avoid carry-over effects, we randomized and counterbalanced the order of sessions between subjects and the minimum period between sessions was 1 week. Participants were not aware of the detailed experimental condition. As it has been introduced in our previous study using SSW qTBS (1), MEP and resting motor threshold (rMT) were recorded with single SSW TMS pulses before (pre-interventional at baseline) as well as at four time points after the end of qTBS (post-qTBS) in PA and AP directions (post 1: 0 min; post 2: 15 min; post 3: 30 min; post 4: 60 min) (Figure 1). Single-pulse SSW TMS produced the same preferential current direction in the precentral gyrus as DSW qTBS (Figures 1, 2A,B). Therefore, the induced current direction in the brain (i.e., AP and PA, respectively) was always the same for evaluation (single-pulse SSW TMS) and intervention (DSW qTBS).

In addition, we compared the reported effects of SSW qTBS and DSW qTBS in the PA and AP directions (supporting information) using a between-subject design (1).

### Electromyographic Recording

The methodological details of electromyographic recording match those reported in our previous study (1). In short, participants were seated comfortably in a chair resting both hands comfortably on a cushion or in their lap to ensure complete relaxation. MEPs were recorded by surface electromyography (EMG) from the non-dominant abductor pollicis brevis (APB) muscle using silver/silver chloride surface electrodes (surface area 263 mm^2^; AMBU, Ballerup, Denmark) mounted according to the bipolar belly-tendon technique. Participants were asked to relax the target muscle throughout the measurement. MEP size was determined by measuring the two highest peaks of opposite polarity and then averaged over 20 trials (20). Trials that differed by more than three times the standard deviation (SD) from the mean were considered outliers and were excluded from the analysis as described previously, which was the case for only one trial (21). The data was bandpass filtered (20–2,000 Hz) and amplified by an Ekida DC universal amplifier (Ekida, Helmstadt, Germany) connected to a Micro 1401 *mk*II data acquisition unit (Cambridge Electronic Design, Cambridge, UK) with a sampling rate of 5 kHz and stored on a personal computer for online visual display and later offline analysis using Signal software version 5 (Cambridge Electronic Design).

### Transcranial Magnetic Stimulation

Procedures of TMS were similar to those introduced previously (1). In detail, the intersection of an eight-shaped stimulation coil (diameter: 100 mm) was centered over the precentral motor hand representation of the non-dominant hand (M1-HAND). The handle pointed in a posterior direction and was lateralized at an angle of ~45° away from the midline.

For single-pulse SSW TMS, the coil was connected to a custom-made magnetic stimulation device (QuattroMag, Munich School of Bioengineering (MSB), Technical University Munich, Munich, Germany) with a biphasic SSW of 160 μs pulse duration (Figure 2B), as reported previously (1, 22). For single-pulse DSW and DSW qTBS, another custom-made magnetic stimulation device (QuattroBurst, MSB, Technical University of Munich, Munich, Germany) with a DSW of the same total pulse duration of 160 μs was used resulting in two concatenated full-sine cycles of 80 μs, respectively (Figure 2A). The reverse of the current direction from PA to AP was performed using a cable that was connected to the coil changing the polarity of each pulse (AP-PA-switch) (1). For single-pulse SSW TMS and SSW qTBS, we refer to PA stimulation when the induced current in the precentral gyrus had a posterior-to-anterior direction, and AP stimulation refers to stimulation producing a preferentially anterior-to-posterior current flow (Figure 2B) (1).

Before each experiment, the optimal site for stimulation (‘hotspot') was determined using single pulse SSW TMS of slightly suprathreshold intensities. The position of the coil was marked with a felt-tip pen. The procedure was repeated prior to DSW qTBS using single pulse DSW TMS to ensure the location of the hotspot for DSW qTBS and to determine the active motor threshold (AMT) for DSW qTBS intensity. Single-pulse SSW TMS used to identify the hotspot and motor thresholds was administered at a frequency of 0.25 Hz. Single-pulse SSW TMS to measure MEP was applied at a pulse repetition rate of 0.1 Hz with a jitter of 15%. Both, rMT and AMT, were determined by a maximum-likelihood threshold-hunting procedure (23) using the TMS Motor Threshold Assessment Tool, version 2 (http://www.clinicalresearcher.org/software.htm). A MEP was defined as a potential larger than 50 μV in peak-to-peak amplitude.

AMT was defined as the lowest intensity that evoked a small response (>100 μV) while participants maintained a slight contraction of the APB of 5–10% of the maximum voluntary contraction, as previously described for quadri-pulse stimulation (1, 9). Voluntary contraction of adequate force was controlled by a manometer. After determination of the motor threshold, we adjusted the stimulator output to elicit mean MEP amplitudes of 800–1,200 μV peak-to-peak (SI1mV) with single pulse SSW TMS for evaluation.

### Double-Sine-Wave (DSW) Quadri-Pulse TBS (qTBS)

DSW qTBS was applied with a double-sine waveform over the precentral motor hand representation of the non-dominant hemisphere, as described previously for SSW qTBS (1). DSW qTBS consisted of bursts with four pulses of the same intensity in intervals of 1.5 ms (~666 Hz). Each burst was separated by 200 ms (5 Hz). A total of 1,440 pulses was delivered in each session with 360 bursts. The stimulus intensity of each pulse was set to 90% DSW AMT (1). Mean stimulation intensity for DSW AP-qTBS at 1.5 ms ISI was 48.00%MSO ± 5.79 and 42.90%MSO ± 6.10 for DSW PA-qTBS at 1.5 ms ISI.

For comparison with SSW qTBS, we evaluated the same participants with the same dataset as reported in our previous study (1).

### Analyses and Statistics

The analyses and statistics of DSW PA-qTBS and DSW AP-qTBS match those reported in our previous manuscript (1). We ensured a sufficient relaxation of the APB by monitoring the electromyographic activity online and by inspecting each MEP sweep again offline. The pre-stimulus time window for determining if MEPs were contaminated by muscle activity was 120 ms. If the electromyographic activity exceeded 0.05 mV, the trial was excluded from further analyses.

All statistical analyses were computed using IBM SPSS Statistics software, version 20.0 (IBM SPSS Statistics Inc., Chicago, IL, USA). Statistical evaluation of DSW qTBS data was performed using repeated-measure analysis of variance (ANOVA) with the inner-subject factors TIME (5 levels: PRE, POST 1, POST 2, POST 3, POST 4) and DIRECTION (2 levels: PA and AP) after the Kolmogorov-Smirnov test revealed no violations of the assumption of normality.

DSW qTBS and SSW qTBS were compared using a rmANOVA with the inner-subject factors TIME (5 levels: PRE, POST 1, POST 2, POST 3, POST 4) and between-subject factor PULSE SHAPE (2 levels: SSW and DSW). No transformations were required.

All statistics were performed using the mean single pulse SSW MEP amplitude of each case computed of 20 MEP trials averaged to a mean, or rMT value (%MSO). Accordingly, the figures display the mean SSW TMS MEP amplitude, or rMT, of all cases. If necessary, we used the Greenhouse-Geisser correction to adjust for violations of sphericity, resulting in adjusted *p*-values based on adjusted degrees of freedom. In the case of significant main effects or interactions, we conducted *post-hoc* two-tailed paired *t*-tests for PRE-POST investigations and for inter-group comparisons, if the same participants took part in the experiment. For inter-group comparisons between DSW qTBS and SSW qTBS data, we computed *post-hoc* two-tailed unpaired *t*-tests. Data was corrected using the Bonferroni correction for multiple comparisons by multiplication of the *p*-values by the number of tests, in this case four. This method was used for MEP and resting motor threshold data. The significance level was set at α = 0.05 for all statistical analyses. All values given are mean group values ± SD, if not indicated otherwise.

## Results

None of the participants reported any adverse events during or after the experiments. Detailed MEP values, standard deviations, and mean TMS intensities (%MSO) in the AP and PA directions, respectively, for each condition at SI1mV are depicted below. We observed no changes in hotspots between single pulse SSW TMS and single pulse DSW TMS which was administered prior to the DSW qTBS in AP and PA directed currents in the precentral motor hand representation.

Comparison of rMT between SSW TMS and DSW TMS recorded prior to DSW qTBS revealed significantly higher threshold values (*p* < 0.01) for DSW TMS in AP and PA directed currents, respectively (Figure 2C).

### Double-Sine Wave (DSW) qTBS at I-Wave Periodicity

Ten volunteers participated in Experiment 1 (DSW PA-qTBS) and Experiment 2 (DSW AP-qTBS), assessing the effect of DSW qTBS at I-wave periodicity with ISI of 1.5 ms. Mean intensity of SSW TMS to target SI1mV was 60.80%MSO ± 14.44 for PA-MEP amplitudes (Experiment 1) and 70.20%MSO ± 11.04 for AP-MEP amplitudes (Experiment 2). rmANOVA of MEP showed a significant main effect of DIRECTION [*F*_(1;9)_ = 18.246, *p* = 0.002] and TIME x DIRECTION interaction [*F*_(4;36)_ = 5.466, *p* = 0.002], but no effect of TIME [*F*_(4;36)_ = 1.738, *p* = 0.163].

**Figure 3:**
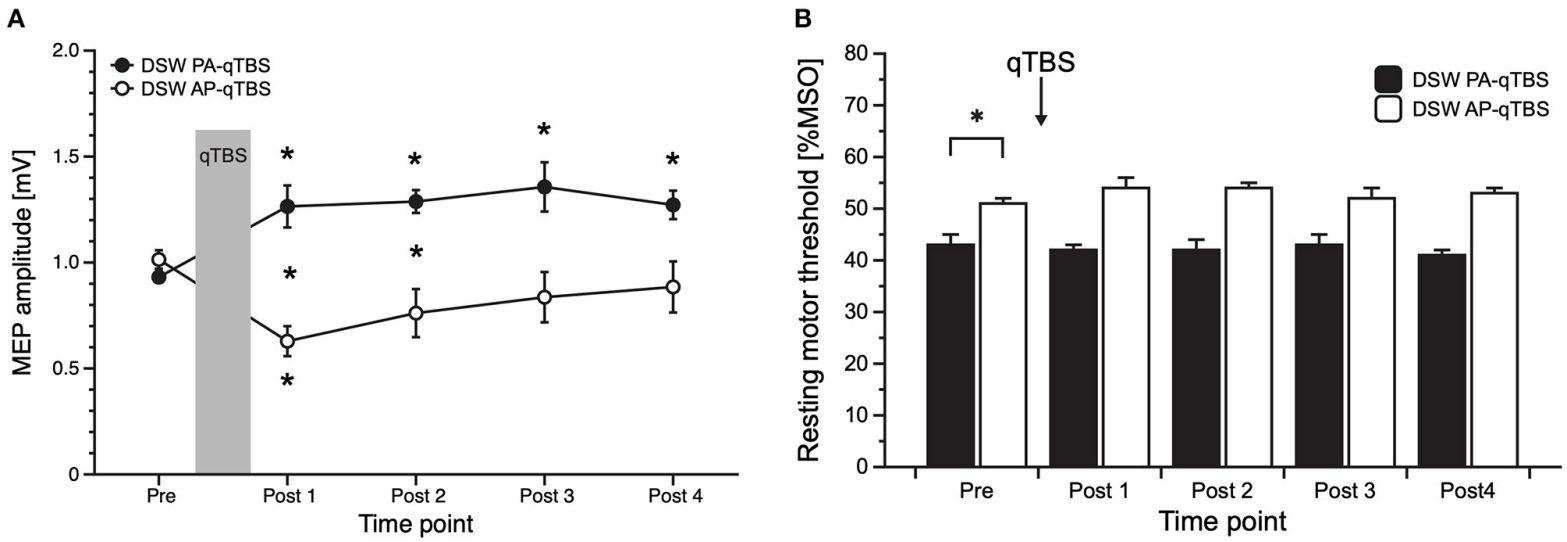
Results of MEP data with corresponding current direction after double-sine-wave (DSW) qTBS in AP and PA directions at ISI of 1.5 ms **(A)** and resting motor threshold following DSW qTBS in AP and PA directions at ISI of 1.5 ms **(B)**. For evaluation, we always used single-sine-wave TMS (SSW TMS) pulses with corresponding current direction as used for qTBS. **(A)** Changing the current flow in M1-HAND from AP to PA led to bidirectional changes in corticospinal excitability with a significant increase of PA-MEP and a significant decrease of AP-MEP, opposite to the bi-directionality observed after SSW qTBS. **(B)** Resting motor threshold of SSW TMS following DSW qTBS in AP and PA directions at ISI of 1.5 ms did not demonstrate significant changes. As observed previously (1), RMT in AP and PA directions significantly differed at pre measurements. Pre: before qTBS, POST1: immediately after qTBS, POST2: 15 min, POST3: 30 min, POST4: 60 min after qTBS. Asterisks indicate significant differences between pre and post measurements (*p* < 0.05). Error bars indicate the standard error of the mean (S.E.M.). qTBS, quadri-pulse theta burst stimulation; PA, posterior-anterior; AP, anterior-posterior; ISI, interstimulus interval; MEP, motor evoked potential; MSO, maximum stimulator output.

PA-MEP amplitudes significantly increased on all time points (*post hoc t*-tests: POST 1: *p* = 0.036; POST 2: *p* = 0.00014; POST 3: *p* = 0.012; POST 4: *p* = 0.004) (Figure 3A).

In the AP direction, mean AP-MEP amplitudes significantly decreased on time point POST 1 (*post hoc t*-test: *p* = 0.004) (Figure 3A).

Between group comparisons revealed significant differences at time points POST 1 (*p* = 0.004) and POST 2 (*p* = 0.008) (Figure 3A).

rmANOVA on rMT data revealed a significant main effect of DIRECTION [*F*_(1;9)_ = 42.178, *p* = 0.000112] but no effect of TIME [*F*_(4;36)_ = 0.809, *p* = 0.528] or TIME x DIRECTION interaction [*F*_(4;36)_ = 2.523, *p* = 0.058]. As expected, and previously observed in the study using SSW qTBS (1), baseline data of SSW TMS rMT prior to DSW qTBS differed significantly (*post hoc t*-test: *p* = 0.000464), with higher thresholds in the AP direction (Figure 3B). Mean SSW TMS AP- and PA-rMT data are presented in Table 1.

**Table 1:** Raw data of motor evoked potential (MEP) in millivolts (mV) and resting motor threshold (rMT) in percent of maximum stimulator output (%MSO) in the respective effective current direction in the brain anterior-posterior (AP) and posterior-anterior (PA) before and after double-sine wave (DSW) qTBS and single-sine wave (SSW) qTBS.

		**Timepoint**					
		**Current direction**	**Pre**	**POST 1**	**POST 2**	**POST 3**	**POST 4**
DSW qTBS	MEP ± SD (mV)	PA	0.93 ± 0.08	1.26 ± 0.31	1.29 ± 0.17	1.36 ± 0.37	1.27 ± 0.24
		AP	1.01 ± 0.14	0.63 ± 0.22	0.76 ± 0.36	0.83 ± 0.38	0.88 ± 0.38
	RMT ± SD (%MSO)	PA	43.50 ± 6.52	42.70 ± 5.60	42.80 ± 6.73	43.30 ± 7.59	41.10 ± 6.10
		AP	51.70 ± 5.23	54.20 ± 8.27	54.80 ± 4.92	52.90 ± 7.26	53.40 ± 5.99
SSW qTBS	MEP ± SD (mV)	PA	0.99 ± 0.14	0.77 ± 0.32	0.81 ± 0.42	0.73 ± 0.42	0.81 ± 0.49
		AP	0.98 ± 0.10	1.20 ± 0.47	1.25 ± 0.48	1.19 ± 0.42	1.41 ± 0.49
	RMT ± SD (%MSO)	PA	50.25 ± 2.53	52.17± 2.78	50.17 ± 2.12	51.83 ± 2.81	52.42 ± 2.93
		AP	59.33 ± 8.40	61.00 ± 11.98	60.58 ± 12.19	61.33 ± 9.74	59.00 ± 10.04

### Single-Sine Wave (SSW) qTBS and Double-Sine Wave (DSW) qTBS in PA-Directed Currents

We compared the effect of single-sine wave (SSW) qTBS and double-sine waves (DSW) qTBS, and analyzed for SSW AP- and PA-qTBS the same dataset as previously published (1). Of these participants (*n* = 12), seven took also part in DSW AP- and PA-qTBS experiments. rmANOVA of raw MEP in PA- and AP-directed currents in the brain were computed.

**Figure 4:**
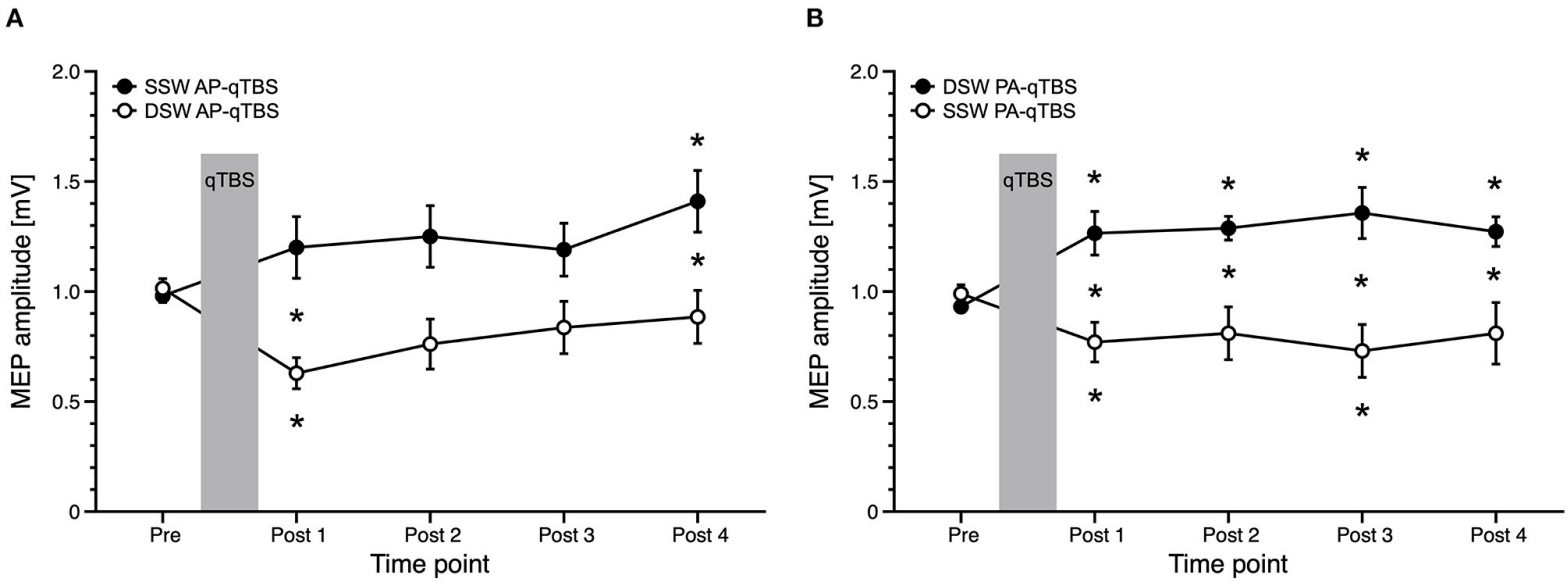
Comparison of MEP data with corresponding current direction after single-sine wave (SSW) and double-sine wave (DSW) qTBS in AP and PA directions, respectively, at ISI of 1.5 ms **(A,B)**. Data are replotted from Figure 3A and from the previous manuscript (1). For evaluation, we always used single-sine wave (SSW) TMS pulses of corresponding current direction. **(A)** Differences of MEP course after SSW AP-qTBS and DSW AP-qTBS demonstrate bidirectional changes in corticomotor excitability in the precentral motor hand representation with a significant increase of AP-MEP after DSW AP-qTBS and a significant decrease of AP-MEP after SSW AP-qTBS. **(B)** Conversely, PA-MEP amplitudes as a sign of changes in corticomotor excitability significantly increased after SSW PA-qTBS and transiently decreased following DSW PA-qTBS. SSW TMS results shown here are the same as previously published (1). Pre: before qTBS, POST1: immediately after qTBS, POST2: 15 min, POST3: 30 min, POST4: 60 min after qTBS. Asterisks indicate significant differences between pre and post measurements or between SSW and DSW measurements within one time point (*p* < 0.05, unpaired *t*-test). Error bars indicate the standard error of the mean (S.E.M.). qTBS, quadri-pulse theta burst stimulation; PA, posterior-anterior; AP, anterior-posterior; ISI, interstimulus interval; MEP, motor evoked potential.

For the PA direction, rmANOVA showed a significant main effect of PULSE SHAPE [*F*_(1;20)_ =14.308, *p* = 0.001] and TIME x PULSE SHAPE interaction [*F*_(1;20)_ = 10.590, *p* = 0.004] with no significant main effects of TIME [*F*_(4;80)_ = 0.561, *p* = 0.692]. Mean single-sine wave MEP amplitudes significantly increased after double-sine wave (DSW) PA-qTBS at all time points, as demonstrated above. Conversely, mean single-sine wave (SSW) MEP amplitude after SSW PA-qTBS significantly decreased at time points POST 1 and POST 3 (*post hoc t*-test: *p* = 0.008, *p* = 0.037, respectively; (Figure 4B). *Post hoc* unpaired *t*-tests revealed a significant difference in SSW MEP amplitudes after SSW and DSW PA-qTBS at all time points (POST 1: *p* = 0.004; POST 2: *p* = 0.012; POST 3: *p* = 0.004; POST 4: *p* = 0.048) (Figure 4B).

After SSW and DSW PA-qTBS, significant main effects on rMT data were observed for PULSE SHAPE [*F*_(1;20)_ = 6.872, *p* = 0.016] but not for TIME [*F*_(4;80)_ = 0.642, *p* = 0.634] or TIME x PULSE SHAPE interaction [*F*_(4;80)_ = 2.469, *p* = 0.051]. *Post hoc* analyses (unpaired *t*-test) revealed no significant differences of baseline data of rMT prior to single- and double-sine PA-qTBS (*p* = 0.058), but between all time points after stimulation (post 1: *p* = 0.013; post 2: *p* = 0.025; post 3: *p* = 0.036; post 4: *p* = 0.006) (Table 1).

### SSW qTBS and DSW qTBS in AP Directed Currents

Comparing AP directed SSW and DSW qTBS effects, rmANOVA revealed a significant TIME x PULSE SHAPE interaction [*F*_(4;80)_ = 3.929, *p* = 0.006] with significant main effects for PULSE SHAPE [*F*_(1;20)_ = 11.070, *p* = 0.003] but not for TIME [*F*_(4;80)_ = 1.839, *p* = 0.129] (Figure 4A). Mean SSW MEP amplitudes after DSW AP-qTBS significantly decreased at time point POST 1 (*p* = 0.004). Mean SSW MEP amplitudes increased after SSW AP-qTBS at time point POST 4 (*post hoc t*-test: *p* = 0.014). Comparing SSW and DSW AP-qTBS, SSW MEP amplitudes were significantly different (*post-hoc* unpaired *t*-test) at time points POST 1 (*p* = 0.008) and POST 4 (*p* = 0.044) (Figure 4A). Detailed MEP values are provided in Table 1.

Analyses of rMT data (rmANOVA) measured with SSW pulses of the same direction revealed no significant main effect of PULSE SHAPE [*F*_(1;20)_ = 3.648, *p* = 0.071], TIME [*F*_(4;80)_ = 1.543, *p* = 0.198] or TIME x PULSE SHAPE [*F*_(4;80)_ = 0.635, *p* = 0.639] after SSW and DSW AP-qTBS.

Comparing the changes in corticospinal excitability between DSW and SSW qTBS of opposite current directions but with the same sign of plasticity (1), we observed a tendency toward a more stable increase in corticospinal excitability after DSW qTBS and a decrease in corticospinal excitability after SSW qTBS (Table 1).

## Discussion

This study extends the findings of previous research on the ability of TBS to alter corticomotor excitability. Using pulses with a double-sine-wave configuration, the present study is the first investigating the after-effects of DSW qTBS and the impact of induced current direction on corticospinal excitability. DSW pulses were applied as quadruple bursts at I-wave periodicity (666 Hz) to preferentially interact with the intracortical circuits in the precentral cortex that project onto the fast-conducting corticospinal neurons. After effects of DSW qTBS on corticospinal excitability were examined using single SSW TMS pulses that produced the same preferential current direction as DSW pulses during qTBS. We found that DSW qTBS at 666 Hz produced lasting changes in corticomotor excitability. The temporal order of phase-related reversals and the resulting order of current reversals in the precentral gyrus determined whether DSW qTBS at I-wave periodicity produced an increase or decrease in corticospinal excitability. If the second component of DSW induced an anterior-to-posterior current in the cortex, DSW AP-qTBS transiently decreased AP-MEP amplitudes. Conversely, DSW PA-qTBS increased PA-MEP amplitudes, if the second component of DSW induced a posterior-to-anterior current in the cortex.

### Double-Sine Wave (DSW) qTBS in PA and AP direction

Biphasic SSW pulses with cycle durations >160 μs are commonly used for TBS. But so far, there is no research on the plasticity inducing effects of TMS pulses that consist of two concatenated full-sine cycles. In this study, we matched the total duration (160 μs) of the DSW pulse to the duration of an SSW pulse (Figures 2A,B). Hence, a single DSW pulse produced five reversals of the induced current direction in the stimulated motor cortex within the 160 μs (Figures 2A,B). Inducing very fast oscillating tissue currents, DSW PA-qTBS at 666 Hz (i.e., at I-wave periodicity) caused a stable increase in PA-MEP amplitudes, whereas DSW AP-qTBS at 666 Hz induced a transient decrease in AP-MEP amplitudes.

These after effects are novel and interesting, but a neurobiological interpretation is challenging and remains in many aspects speculative. Previous electrophysiological TBS studies are of little help as they used biphasic SSW pulses with longer single-cycle duration. Given the short cycle length of our DSW pulse, polarity reversals occurred at a faster rate and thus, the rise times of the electrical field during a single pulse component were steeper, but shorter. Therefore, the biophysical effects of a given AP or PA component of the DSW pulse can be expected to differ substantially from the effects evoked by the AP or PA component of a standard SSW pulse. Due to the shorter duration, the depolarizing and hyperpolarizing effects of the DSW components on the axonal membrane may interact. The earlier components of the DSW pulse may enhance or attenuate the likelihood of the later components of the DSW pulse to alter the membrane state and to evoke changes in corticomotor excitability by eliciting action potentials in the targeted cortex region with DSW qTBS.

Computational models and experimental observations suggest that the effect of electrical stimulation with reversed double pulses on the probability to elicit an action potential depends on the sequence of polarity within a pulse and on the value of the membrane potential at the time of stimulation (16). In a hyperpolarized membrane state, an initial negative double-sine shaped pulse is more effective in generating action potentials in animal models, while in a depolarized membrane, an initially positive pulse is more effective (16). It has been argued, using biphasic pulses, that the initially negative short falling component of the pulse leads to a hyperpolarization of the nerve membrane and removes a small degree of the resting level of sodium channel inactivation (24). This, in turn, renders the following long rising component more effective in depolarizing the nerve membrane and eliciting action potentials (24). The long rising or falling second component in biphasic pulses dominates the depolarizing or hyperpolarizing effect which is primed by the initial current of the short component (24). In this context, a hyperpolarization of the nerve has been shown to increase the availability of sodium channels during the subsequent depolarizing component of the pulse and triggers action potentials elicited by the pulse (24). However, the situation may be much more complex using DSW pulses due to the many reversals of the current. In this case, we may only speculate about the mechanisms and further investigations of the detailed neurophysiological mechanisms are needed, as these hypotheses of different membrane states are not directly supported by our experiments.

The preceding components of DSW used in the present study may have influenced the responsiveness of the cortical target structures (axons) to the depolarizing or hyperpolarizing effects of the tissue current induced by later phases of a DSW pulse, while the priming effect of a preceding hyperpolarization may explain the sign of the aftereffects induced by DSW qTBS. We speculate that this leads to an increase in corticomotor excitability after DSW PA-qTBS, which is explained by a higher efficacy of this double-sine shaped pulse to elicit action potentials.

In contrast, the transient decrease in corticomotor excitability after DSW AP-qTBS may be due to the hyperpolarization of predominantly AP directed currents. Thus, DSW AP-qTBS may generate action potentials in fewer neurons (16, 24–26). However, we remain very speculative as the detailed cellular mechanisms have not been directly investigated (e.g., on single neurons).

Another explanation of the effects may be that each DSW pulse of the respective current direction results in a greater net activation of AP- and PA-directed currents (here, DSW AP-qTBS and DSW PA-qTBS), respectively. Comparing the results of SSW qTBS and DSW qTBS of the same preferential current direction in the brain, we demonstrated an opposite sign of plasticity (Figures 4A,B). The idea of the same effective current direction in the brain (i.e., AP and PA) is supported by the directional dependency of rMT values, which is in agreement with our hypothesis, that DSW AP-qTBS mainly induces an AP-directed current in the brain while DSW PA-qTBS mainly induces a PA-directed current flow in the brain (Figure 2C). Hence, we assume further mechanisms of DSW pulses to be responsible for the bidirectional effects of DSW qTBS on corticospinal excitability.

The interpretation of our findings is further complicated by the assumption that AP and PA currents in the precentral gyrus may produce preferential excitation of different sets of cortical neurons (27). Implementing realistic models of cortical neurons, a recent modeling study of TMS-induced electrical fields in the precentral gyrus identified intracortical axonal terminations in the superficial crown and lip regions as primary stimulation target sites (28). Relevant to our study, varying the induced current direction (AP vs. PA) caused an anterior-posterior shift in precentral activation for both monophasic and biphasic pulse (SSW) configurations (28). This leads to a preferential excitation of differently oriented axon terminals in the anterior (AP current) or posterior (PA current) lip regions of the precentral gyrus. Since cortical neurons in the precentral crown display marked regional differences in their sensitivity to fire in response to the rapidly changing AP or PA-directed currents, we argue that DSW PA-qTBS and AP-qTBS targeted spatially distinct sets of cortical neurons in the precentral cortex. This may be an important additional cause for why DSW PA-qTBS induced opposite effects on corticomotor excitability compared to AP-qTBS.

### Double-Sine-Wave (DSW) qTBS and Single-Sine-Wave (SSW) qTBS

The direction of plasticity produced by DSW qTBS at I-wave periodicity was opposite in sign compared to the bidirectional excitability changes that we had previously observed after SSW qTBS at I-wave periodicity. This was the case when we compared the SSW qTBS conditions (applied in our previous study) and the DSW qTBS conditions (applied in this study) of the same preferential current direction in the precentral motor hand representation. We found an increase in corticomotor excitability after DSW PA-qTBS and a decrease after SSW PA-qTBS, while DSW AP-qTBS led to a decrease and SSW AP-qTBS to an increase (Figure 4). Comparing the changes in corticospinal excitability between DSW and SSW qTBS of opposite current direction but with same sign of plasticity (1), the increase in corticospinal excitability was slightly more evident after DSW PA-qTBS than SSW AP-qTBS indicating DSW qTBS is more effective.

The burst frequency (666 Hz) for both, DSW qTBS and SSW qTBS, was chosen to interact with I-wave periodicity, for instance by modifying the fidelity of spike timing mechanisms for single-sine qTBS (1, 29). We consider a differential effect of DSW and SSW pulses on the high-fidelity spike-timing mechanisms at I-wave periodicity to be unlikely, given the very high frequency of the alternating tissue current in the kHz range. Rather, we propose that the aforementioned mechanisms of change in membrane states and action potential generation account for the differences in current-orientation specific effects. However, we may not exclude a rather simple explanation that DSW PA-qTBS has an AP-like stimulation effect and, consequently, DSW AP-qTBS has a PA-like stimulation effect. Although this seems to be unlikely since an evaluation in the opposite current direction of SSW TMS to DSW qTBS demonstrated no changes in corticospinal excitability (Supplementary Material). Moreover, I-wave excitability appears to play a central role in modulating corticospinal excitability (30). Regardless of what the underlying mechanisms may be, the results of our qTBS work highlights the pulse configuration as an important variable of TMS interventions. Our observation motivates future research examining, in detail, how the number of cycles and cycle length of SSW, DSW, and poly-sine wave pulses influence the efficacy of inducing action potentials in axonal structures in the targeted cortex. Such research may inform future attempts to optimize the pulse configurations used for interventional rTMS in a therapeutic setting.

### Differences to Previous Findings and Safety Issues

In a therapeutic setting, the motivation for applying rTMS is to induce stable changes in cortical excitability and function (31). Here, we introduce a modified version of the existing qTBS protocol using a novel pulse configuration which may draw on mechanisms of neuronal excitation that could not be investigated previously with conventional either monophasic or biphasic single-sine-wave pulse configurations. Comparing our findings to other TMS studies that investigated corticospinal plasticity in humans, the increase or decrease in corticomotor excitability resembles previously reported LTP- and LTD-like plasticity (8). Yet, the mechanisms that determine the induction of action potentials with DSW pulses remain to be explored. Further investigations using DSW to better understand the mechanisms of the new stimulation protocol are needed.

Our previous findings demonstrated that using pulses that consisted of multiple sine cycles is more effective in exciting corticospinal output neurons in the precentral motor hand representation than single-sine cycles (14). Here, we observed a slightly higher threshold for DSW pulses of app. 10 %MSO as compared to our previous findings (14). Since we did not use the same stimulation device, this difference may be attributed to the technical pattern because of different capacitors and repetition of the pulses or neurophysiological differences in chronaxie and rheobase. Additional experiments comparing SSW pulses of 80 μs and DSW pulses of 160 μs may provide additional insights.

Furthermore, it is worth mentioning that the directional dependency of rMT values is in line with our hypothesis, that the preferential currents in the brain induced by DSW stimulation cause higher rMT values for AP-directed currents than for PA-directed currents. This may support the idea of predominantly PA- and AP-directed tissue currents and activation as illustrated in Figure 2C.

As a limitation, we evaluated changes in corticospinal excitability only by SSW single-pulse TMS. As we used amplitudes with an intensity to target 1 mV (SI1mV) and rMT of DSW TMS was high (Figure 2C) we were unable to target SI1mV by DSW single-pulse TMS. Moreover, we limited the stimuli count before experiments to avoid occlusion of possible plasticity effects in human primary motor cortex (21). The study is further limited by a rather small numbers of participants. However, we tried to minimize any confounding factors by choosing an intra-subject design for DSW qTBS (and for SSW qTBS). Even then, DSW qTBS has demonstrated clear (bidirectional) effects on corticospinal plasticity.

In this study, DSW pulses were used for the first time for ultra-high-frequent qTBS. This raises the question whether the novel protocol is equally as safe as previously introduced rTMS protocols. We performed the intervention with stimulation intensities below active motor thresholds according to existing safety guidelines (18). The protocol was well-tolerated by all participants with no adverse effects or spread of excitation to neighboring muscles during stimulation. However, further studies are needed to confirm the safety and clinical use of patterned rTMS protocols using DSW pulses.

## Conclusions

We demonstrated bi-directional changes in corticospinal excitability after ultra-high frequency DSW qTBS over human precentral motor hand representation. The induced current direction in the brain determined the sign of plasticity of DSW qTBS at ISI that target I-wave periodicity (i.e., 666 Hz). Bi-directional effects were opposite to those observed after SSW qTBS in the respective current direction. The results may be explained by the effects of alternating medium frequency current at axonal membranes. Our findings may be of relevance when designing new and effective non-invasive TMS protocols for research and therapeutic purposes and may provide new insights into mechanisms of corticospinal excitability in the human precentral gyrus. The results of this study may offer new opportunities for short non-invasive brain stimulation protocols that are especially suited for transcranial magnetic stimulation in children and patients with neurological or neurodevelopmental disorders.

## Data Availability Statement

The original contributions presented in the study are included in the article/Supplementary Material, further inquiries can be directed to the corresponding authors.

## Ethics Statement

The studies involving human participants were reviewed and approved by Ethics Committee of the Technical University of Munich, Faculty of Medicine. The patients/participants provided their written informed consent to participate in this study.

## Author Contributions

NJ, BG, HS, and VM conceptualized and designed the study and supervised the work. NJ, BG, NG, AK, EA, and JF were involved in data acquisition, including patient recruitment, and data analysis. NJ, BG, NG, AK, and JF were involved in the analysis and interpretation of the data. NJ wrote the first draft of the manuscript. All authors approved the final manuscript.

## Conflict of Interest

HS has received honoraria as speaker from Sanofi Genzyme, Denmark and Novartis, Denmark, as a consultant from Sanofi Genzyme, Denmark and as editor-in-chief (NeuroImage Clinical) and senior editor (NeuroImage) from Elsevier Publishers, Amsterdam, Netherlands. He has received royalties as book editor from Springer Publishers, Stuttgart, Germany and Gyldendal Publishers, Copenhagen, Denmark. The remaining authors declare that the research was conducted in the absence of any commercial or financial relationships that could be construed as a potential conflict of interest.
