# Supplementary material for: Double-Sine-Wave Quadri-Pulse Theta Burst Stimulation of Precentral Motor Hand Representation Induces Bidirectional Changes in Corticomotor Excitability
Source: Front Neurol. 2021 Jun 28;12:673560. doi: 10.3389/fneur.2021.673560 (PMC8273174; doi:10.3389/fneur.2021.673560)
Supplement: Supplementary file 1 [file Data_Sheet_1.docx]

Supplementary Material

# Supplementary Results and Data

In a subset of participants, we compared the effect of double-sine wave (DSW) in AP- or PA direction with the opposite current direction of single-sine wave (SSW) AP- or PA-TMS, i.e. DSW AP-qTBS and SSW PA-TMS, and DSW PA-qTBS and SSW AP-TMS. Seven participants (4 women, 3 men) aged 22 to 37 years (median age 23.0 years; SD 5.40) underwent the DSW AP-qTBS and SSW PA-TMS experiment, and four (2 women, 2 men) aged 22 to 24 years (median age 23.0 years; SD 0.82) took part the DSW PA-qTBS and SSW AP-TMS experiment. The methods are described in detail in the main manuscript. rmANOVA of raw MEP in PA- and AP-directed currents in the brain revealed no significant main effects (TIME, PULSE SHAPE and TIME x PULSE SHAPE) in both experiments (supplementary Figure 1). No changes in resting motor thresholds have been observed as well.

# Supplementary Figure


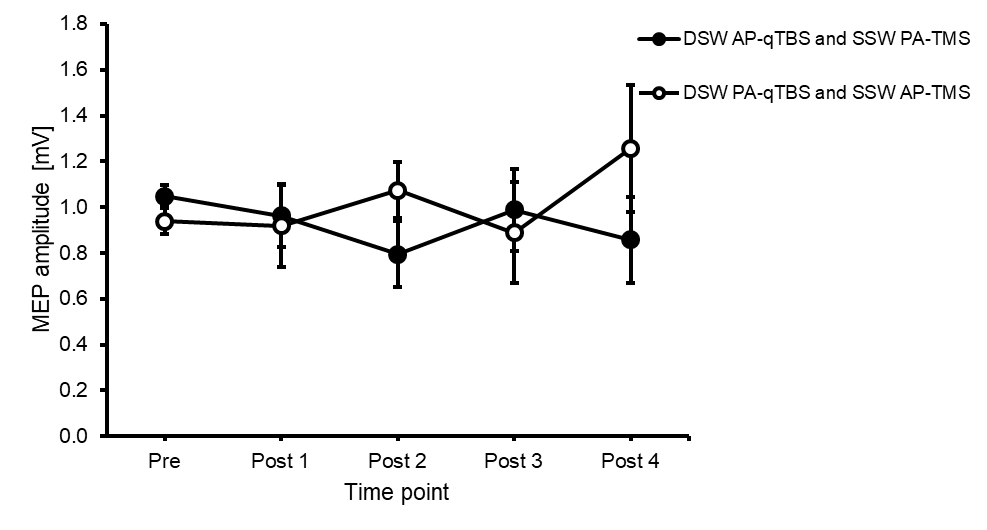


**Supplementary Figure 1:** Comparison of MEP data after double-sine wave (DSW) AP- and PA-qTBS at ISI of 1.5 ms evaluated in the opposite current direction of single-sine wave (SSW) AP- or PA-TMS, i.e. DSW AP-qTBS and SSW PA-TMS, and DSW PA-qTBS and SSW AP-TMS, respectively. For evaluation, we always used single-sine wave (SSW) TMS pulses of opposite current direction. Differences of MEP course after DSW AP-qTBS and SSW PA-TMS, and DSW PA-qTBS and SSW AP-TMS demonstrate no changes in corticomotor excitability in the precentral motor hand representation. Pre: before qTBS, POST1: immediately after qTBS, POST2: 15 min, POST3: 30 min, POST4: 60 min after qTBS. Error bars indicate the standard error of the mean (S.E.M.). qTBS: quadri-pulse theta burst stimulation; PA: posterior-anterior; AP: anterior-posterior; DSW: souble-sine wave; SSW: single-sine wave; MEP: motor evoked potential.
